# Body image, self-esteem, and quality of life in patients with primary malignant bone tumors

**DOI:** 10.1007/s00402-019-03205-8

**Published:** 2019-05-24

**Authors:** Lukas A. Holzer, Nicolas Huyer, Jörg Friesenbichler, Andreas Leithner

**Affiliations:** 1 Department of Orthopaedics and Trauma, Medical University Graz, Auenbruggerplatz 5, 8036 Graz, Austria; 2 AUVA Trauma Center Klagenfurt, Klagenfurt am Wörthersee, Austria

**Keywords:** Osteosarcoma, Ewing-Sarcoma, Quality of life, Self-esteem, Body image

## Abstract

**Background:**

Patients with primary malignant bone tumors are facing different challenges in their everyday lives due to improved treatment and prolonged survival. This raises the question whether and to what extent their quality of life, body image, and self-esteem is affected by their disease. The aim of this retrospective study was to analyze the quality of life, body image and self-esteem of patients with primary malignant bone tumors compared to a healthy control group.

**Methods:**

A total of 56 patients (39 male, 17 female; average age 33.8 [± 14.29] years) who were treated with either osteosarcoma or Ewing-Sarcoma at the authors’ institution between Jan 1989 and May 2015 were included into the study (mean follow-up: 9.1  ± 6.6] years). The control group consisted of 58 (average age 24.4 [± 3.1] years, 31 male, 27 female) healthy medical students. Standardized questionnaires were used to assess quality of life (SF-36), body image (MBSRQ) and self-esteem (RSE-scale). Student’s *t* test were used for statistical analysis.

**Results:**

Quality of life (SF-36) (in physical categories) and body image (MBSRQ) was significantly lower in patients with primary malignant bone tumors compared to healthy cohort (*p* < 0.001). Self-esteem was not affected i n patients and did not show any difference compared to control group (23.96 vs. 24.00).

**Discussion:**

Physical categories of quality of life and body image sensation of patients with primary malignant bone tumors are worse compared healthy controls. However, self-esteem does not seem to be affected by the condition and its management. Patients can be encouraged about this at the time of diagnosis of a primary malignant bone tumor.

## Introduction

Primary malignant bone tumors are among the rarest types of cancer [1]. In the United States, these entities are responsible for only 0.2% of all malignant neoplasia diagnosed per year [2]. Two of the most common primary malignant bone tumors are the osteosarcoma (OS) (35%) and the Ewing-Sarcoma (ES) (16%) [1]. OS can develop at any age, but like ES, it is particularly common in children and young adults and makes up a significant percentage (up to 8%) of cancer types in these age groups [3].

The treatment of primary malignant bone tumors consists of surgical resection, chemotherapy and radiotherapy in cases of ES. Recent advances in the treatment have significantly improved the survival of patients who suffer from these tumors. Nonetheless, the 5-year survival is still very low (4–87% for OS, 20–70% for ES) [4, 5].

Surviving patients are facing different problems in their everyday lives. Apart from the biological aspects of these tumors, psychological and social challenges should not be forgotten.

Currently, the literature referring to social and psychological effects in patients with primary malignant bone tumors is limited. Some studies aimed to evaluate QoL in patients with primary bone tumors [6–8]. Other studies aimed to assess the body image of tumor patients [9, 10]. One study analyzed the self-esteem of cancer patients [11].

The aim of this study was to compare QoL, body image and self- esteem of patients with primary malignant bone tumors (osteosarcoma and Ewing-Sarcoma) to a healthy control group. Four different self-administered questionnaires were used. The Short-Form 36 (SF-36) questionnaire was used to assess QoL, the Multi-Dimensional Body-Self Relation Questionnaire (MBSRQ) to assess body image and the Rosenberg Self-Esteem Scale (RSE) to assess self-esteem. Functionality of the affected limb was evaluated using the Musculoskeletal Tumor Society Score (MSTS).

## Materials and methods

205 patients (121 OS, 84 ES; 124 male, 81 female) were treated at the authors’ institution between Jan 1989 and May 2015. 112 (54.6%) surviving patients (69 male, 43 female) were invited to participate in the study. They received a letter with a description and an invitation to the study. A total of 56 (50%) patients (39 male, 17 female, mean age 33.9 [± 14.3] years) with osteosarcoma and Ewing-Sarcoma could be contacted and were included in the study (mean follow-up: 9.1 [± 6.6] years, range of follow-up 8 months–22 years). Inclusion criteria were the ability to speak German fluently and the ability to read and write ( to fill in the questionnaires). Exclusion criteria were ages under 16 years and over 85 years. There were 34 (60.7%) osteosarcoma patients and 22 (39.3%) Ewing-Sarcoma patients. The demographical data of all patients can be seen in Table 1.

**Table 1:** Demographic data of patients and controls

		Patient		Control
		Osteosarcoma	Ewing-Sarcoma	
*N*		56		58
		34 (60.7%)	22 (39.3%)	
Age (in years)	MV (STD)	33.9 (± 14.3)		24.4 (± 3.1)
		35.5 (± 16.5)	31.1 (± 9.6)	
Male (%)		39 (69.6%)		31 (53.5%)
		22 (64.7%)	17 (77.3%)	
Female (%)		17 (30.4%)		27 (46.5%)
		12 (35.3%)	5 (22.7%)	
Years since diagnose	MV (SDT)	9.1 (± 6.6)		
		8.2 (± 6.0)	10.3 (± 7.4)	

After the invitation letters were sent to the patients, they were contacted by phone and asked to give their personal email address. Those patients who could not be reached, received five attempts by phone to collect the data. All patients that could be contacted took part in this study. Using the online survey platform http://www.surveymonkey.com (SurveyMonkey^©^ Palo Alto, California, USA) a questionnaire was designed and sent to all participants. Forty-nine patients filled in the online questionnaire, seven patients could not name an email address and were, therefore, interviewed by telephone. Patients’ demographic and clinical data were extracted from the tumor database of the authors’ institution including age (in years), sex (male or female), medical history (diagnosis, conducted treatment, localization of tumor). According to their clinical and demographic data patients were divided into different groups: (1) patients with primary tumors located in the lower limb (including pelvis); (2) patients with primary tumors located in the upper limb (including shoulder and clavicle); and (3) patients with primary tumors located in the trunk. Furthermore, patients were divided into four groups based on whether they did or did not receive chemotherapy, and whether they did or did not receive radiotherapy. For patient's characteristics see Table 2.

**Table 2:** Demographic data of patient groups

		Lower limb		Upper limb	Trunk	Chemotherapy		Radiotherapy	
		Salivation	Amputation			Yes	No	Yes	No
*N* (%)		42 (75.0%)		7 (12.5%)	7 (12.5%)	48 (85.7%)	8 (14.3%)	14 (25.0%)	42 (75.0%)
		37 (66.1%)	5 (8.9%)						
OS (%)		30 (71.4%)		3 (42.9%)	1 (14.3%)	27 (56.2%)	7 (87.5%)	3 (21.4%)	36 (85.7%)
		27 (73.0%)	3 (60.0%)						
ES (%)		12 (28.6%)		4 (57.1%)	6 (85.7%)	21 (43.8%)	1 (12.5%)	11 (78.6%)	6 (14.3%)
		10 (27.0%)	2 (40.0%)						
Age (in years)	MV (SD)	33.1 (± 6.8)		33.0 (± 18.5)	39.0 (± 10.4)	31.6 (± 11.5)	47.5 (± 21.7)	32.6 (± 9.9)	34.1 (± 15.7)
		32.7 (± 13.5)	35.6 (± 20.2)						
Male (%)		27 (64.3%)		6 (85.7%)	6 (85.7%)	36 (75.0%)	3 (37.5%)	11 (78.6%)	28 (66.7%)
		24 (64.9)	3 (60.0%)						
Female (%)		15 (35.7%)		1 (14.3%)	1 (14.3%)	12 (25.0%)	5 (62.5%)	3 (21.4%)	14 (33.3%)
		13 (35.1%)	2 (40.0%)						
Since diag (in years)	MV (SD)	9.75 (± 6.8)		5.3 (± 4.8)	8.5 ( ± 6.1)	9.8 (± 9.6)	4.9 (± 5.2)	11.0 (± 6.8)	8.4 (± 6.5)
		9.9 (± 6.9)	8.7 ( ± 7.3)						

Medical students of the Medical University Graz were recruited as healthy controls. The same inclusion/exclusion criteria were applied as above. A total of 58 (31 male, 27 female, mean age 24.4 [± − 3.1] years) students were enrolled into the study. For demographic data of healthy controls see Table 1.

### Questionnaires

To evaluate the quality of life, self-esteem and body image four different questionnaires were used: (1) Short-Form (36) Health Survey (SF-36) for QoL; (2) Rosenberg Self-esteem Scale (RSE) for self-esteem; (3) Multidimensional Body-Self Relations Questionnaire (MBSRQ) for body image; and (4) Musculoskeletal Tumor Society Score (MSTS) for function.

#### Short-Form (36) Heath Survey (SF-36)

The SF-36 is a questionnaire designed to assess generic health using 36 items, that cover eight health-related quality of life domains: physical functioning (PF), role limitation due to physical difficulties (RF), bodily pain (BP), general health (GH), vitality (V), social function (SF), role limitation (RE) due to emotional difficulties and mental health (MH). Each domain is scored, summed and transformed differently. The result is a score from 0 (worst quality of life) to 100 (best quality of life) for each domain. It is a standardized questionnaire used globally, that is not specific to age, disease or treatment group [12].

#### Rosenberg Self-Esteem Scale (RSE)

This 10 item-containing self-reporting questionnaire is used to assess general personal self-esteem in one summary scale. The RSE uses a four point Likert scale to rate each item (0 points for strongly agree and 3 points for strongly disagree). The questionnaire adds up to a score from 0 to 30 points, whereby high scores indicate high self-esteem [13, 14].

#### Multidimensional Body-Self Relations Questionnaire (MBSRQ)

The German version of the MBSRQ is a 71-item, self-reported questionnaire to assess ones’ body image construction in two dimensions: “evaluation” and “orientation”. It is divided into seven different subscales: “health evaluation” (HE) measuring feelings of physical health; “health orientation” (HO) measuring extent of investment to maintain health; “appearance evaluation” (AE) focusing on physical attractiveness and satisfaction of ones’ appearance; “appearance orientation” (AO) evaluating the amount of work put in ones’ look; “fitness evaluation” (FE) measuring feelings of being physical fit; “fitness orientation” (FO) assessing the extent of investment of being physically fit; and “illness orientation” (IO) focusing on the extent of reaction when becoming ill. Moreover, 17 items assess ones’ satisfaction to certain body areas (SB), the estimation of ones’ weight and fear of obesity. Each item is answered on a 5-point Likert scale ranging from 1 (“definitely disagree”) to 5 (“definitely agree”). The ten different subscales are scored with a computer- assisted formula [15, 16].

#### Musculoskeletal Tumor Society Score (MSTS)

The questionnaire published by the Musculoskeletal Tumor Society in 1989 is a widely used tool to assess functions of limbs after surgical resections of musculoskeletal tumors. It contains six questions evaluating function on a scale from 0 (poor) to 5 (very good). Scores are summed and give a maximum of 30 points, where high scores indicate a good function [17].

### Statistics

All data was managed with Microsoft^©^ Excel (Microsoft^©^ Excel for Mac 2011 Version 14.1.0, Microsoft Corporation, Redmond, USA). To identify any significant differences between patients and controls, Student’s *t* test was used. A *p* value of < 0.05 was considered significant.

### Conflict of interests

Each author certifies that he or she has no commercial associations (e.g., consultancies, stock ownership, equity interest, patent/licensing arrangements, etc.) that might pose a conflict of interest in connection with the submitted article.

### Ethical review committee statement

This study was performed in accordance with the Helsinki Declaration and approved by the institutional review board (EK 27-011 14/15). All participants gave informed consent and had the opportunity to leave the study at any time.

## Results

The results of the questionnaires for each different group can be seen in Table 3.

**Table 3:** Results of questionnaires

		Patients						Control		
		All	Male	Female	Lower extremity	Upper extremity	Trunk	All	Male	Female
SF36	PF	66.79 (± 28.16)	67.95 (± 28.04)	64.12 (± 29.11)	63.45 (± 29.16)	65.71 (± 27.6)	87.86 (± 9.94)	98.02 (± 8.68)	99.44 (± 1.6)	98.75 (± 2.24)
	RF	62.05 (± 42.64)	66.67 (± 41.49)	51.47 (± 44.61)	58.93 (± 43.06)	57.14 (± 53.45)	85.71 (± 19.67)	97.84 ( ± 10.77)	100 (± 0)	93.75 (± 19.36)
	BP	69.91 (± 28.05)	71.97 (± 27.53)	65.18 (± 29.5)	69.21 (± 27.63)	70.86 (± 36.64)	73.14 (± 25.26)	91.52 (± 13.87)	91.74 (± 14.82)	88.56 (± 14.81)
	GH	68.20 (± 20.89)	70.64 (± 19.32)	62.59 (± 23.79)	66.14 (± 20.94)	75.14 (± 22.2)	73.57 (± 19.81)	78.59 (± 16.75)	77.07 (± 18.33)	76.75 (± 18.42)
	V	59.29 (± 19.13)	61.67 (± 18.26)	53.82 (± 20.5)	57.38 (± 17.61)	60 (± 29.72)	70 (± 13.84)	64.66 (± 13.6)	63.33 (± 14.21)	65.63 (± 13.89)
	SF	81.92 (± 26.75)	84.29 (± 24.12)	76.47 (± 32.14)	82.14 (± 27.49)	73.21 (± 29.25)	89.29 (± 19.67)	89.87 (± 15.08)	90.28 (± 14.84)	86.72 (± 17.95)
	ER	79.76 (± 35.78)	85.47 (± 32.26)	66.67 (± 40.82)	81.75 (± 34.69)	71.43 (± 40.5)	76.19 (± 41.79)	90.8 (± 24.82)	96.3 (± 19.25)	79.16 (± 34.16)
	MH	76.57 (± 17.11)	77.74 (± 15.35)	73.88 (± 20.89)	75.62 (± 17.4)	69.14 (± 17.24)	89.71 (± 6.87)	80.55 (± 12.64)	79.41 (± 13.48)	77.5 (± 13.14)
MBSRQ	AE	3.58 (± 0.77)	3.67 (± 0.75)	3.38 (± 0.82)	3.56 (± 0.84)	3.41 (± 0.54)	3.86 (± 0.52)	3.99 (± 0.48)	4.02 (± 0.46)	3.91 (± 0.51)
	AO	3.19 (± 0.65)	3.04 (± 0.53)	3.55 (± 0.77)	3.26 (± 0.65)	3.36 (± 0.4)	2.67 (± 0.65)	3.32 (± 0.62)	3.23 (± 0.6)	3.42 (± 0.62)
	FE	3.64 (± 0.76)	3.78 (± 0.78)	3.29 (± 0.6)	3.6 (± 0.74)	3.54 (± 0.85)	3.97 (± 0.78)	4.05 (± 0.47)	4.1 (± 0.4)	3.9 (± 0.58)
	FO	3.33 (± 0.88)	3.5 (± 0.89)	2.94 (± 0.75)	3.3 (± 0.86)	3.49 (± 1.09)	3.34 (± 0.94)	4.1 (± 0.65)	4.03 (± 0.67)	4.22 (± 0.46)
	HE	3.63 (± 0.77)	3.71 (± 0.77)	3.43 (± 0.76)	3.55 (± 0.71)	3.6 (± 0.99)	4.14 (± 0.76)	4.07 (± 0.62)	4.08 (± 0.65)	3.93 (± 0.67)
	HO	3.56 (± 0.59)	3.39 (± 0.58)	3.93 (± 0.42)	3.5 (± 0.54)	3.54 (± 0.83)	3.93 (± 0.57)	3.77 (± 0.52)	3.66 (± 0.55)	3.95 (± 0.35)
	IO	3.18 (± 0.77)	3.17 (± 0.69)	3.2 (± 0.94)	3.08 (± 0.8)	3.71 (± 0.49)	3.26 (± 0.64)	3.08 (± 0.74)	3.14 (± 0.79)	3.18 (± 0.64)
	SB	3.59 (± 0.74)	3.65 (± 0.77)	3.43 (± 0.66)	3.62 (± 0.78)	3.41 (± 0.55)	3.56 (± 0.73)	3.85 (± 0.58)	3.84 (± 0.6)	3.87 (± 0.34)
	∑	3.41 (± 0.44)	3.43 (± 0.48)	3.38 (± 0.34)	3.39 (± 0.43)	3.49 (± 0.53)	3.48 (± 0.45)	3.78 (± 0.28)	3.75 (± 0.3)	3.81 (± 0.26)
Rosenberg		23.96 (± 5.60)	23.97 (± 5.35)	23.94 (± 6.3)	23.45 (± 6.15)	23.43 (± 1.9)	27.57 (± 2.76)	24.00 (± 5.32)	24.63 (± 4.29)	22.81 (± 7.12)
MSTS		17.60 (± 7.39)	18.66 (± 6.66)	15.5 (± 8.5)	17.32 (± 7.62)	19.29 (± 6.05)				

### QoL

Regarding the QoL and the SF-36, significantly lower scores for patients compared to control were found in the subscales assessing physical health: PF, RF, BP and GH. No significant differences were detected in the categories evaluating the psychological health (Table 4).

**Table 4:** Statistical comparison of different groups

		Patients vs. control	Male vs. male	Female vs. female	Male patients vs. female patients	Lower extremity vs. control	Upper extremity vs. control	Trunk vs. control	Lower extremity vs. upper extremity	Upper extremity vs. trunk	Lower extremity vs. trunk
SF36	PF	*/< 0.001	*/< 0.001	*/< 0.001	ns/0.650	*/< 0.001	*/0.022	*/0.035	ns/0.847	ns/0.083	*/< 0.001
	RF	*/< 0.001	*/< 0.001	*/0.001	ns/0.359	*/< 0.001	ns/0.092	ns/0.157	ns/0.935	ns/0.223	*/0.015
	BP	*/< 0.001	*/0.001	*/0.003	ns/0.425	*/< 0.001	ns/0.207	ns/0.104	ns/0.913	ns/0.894	ns/0.716
	GH	*/0.004	ns/0.072	*/0.023	ns/0.229	*/0.013	ns/0.843	ns/0.541	ns/0.347	ns/0.891	ns/0.632
	V	ns/0.088	ns/0.634	*/0.039	ns/0.185	ns/0.052	ns/0.726	ns/0.364	ns/0.828	ns/0.442	ns/0.059
	SF	ns/0.055	ns/0.172	ns/0.153	ns/0.377	ns/0.168	ns/0.209	ns/0.942	ns/0.495	ns/0.254	ns/0.423
	ER	ns/0.059	ns/0.159	ns/0.092	ns/0.105	ns/0.228	ns/0.282	ns/0.397	ns/0.543	ns/0.832	ns/0.748
	MH	ns/0.162	ns/0.605	ns/0.174	ns/0.499	ns/0.362	ns/0.203	*/0.012	ns/0.384	*/0.019	*/0.001
MBSRQ	AE	*/0.001	*/0.015	*/0.019	ns/0.217	*/0.007	*/0.032	ns/0.555	ns/0.532	ns/0.141	ns/0.238
	AO	ns/0.302	ns/0.117	ns/0.448	*/0.019	ns/0.757	ns/0.748	*/0.039	ns/0.589	*/0.038	*/0.009
	FE	*/< 0.001	*/0.039	*/< 0.001	*/0.014	*/0.002	ns/0.189	ns/0.806	ns/0.882	ns/0.344	ns/0.268
	FO	*/< 0.001	*/0.017	*/< 0.001	*/0.019	*/< 0.001	ns/0.193	ns/0.076	ns/0.672	ns/0.782	ns/0.925
	HE	*/< 0.001	*/0.010	*/0.017	ns/0.211	*/0.002	ns/0.306	ns/0.826	ns/0.906	ns/0.269	ns/0.089
	HO	*/0.046	ns/0.076	ns/0.806	*/< 0,001	*/0.020	ns/0.504	ns/0.496	ns/0.909	ns/0.326	ns/0.098
	IO	ns/0.498	ns/0.848	ns/0.512	ns/0.905	ns/0.642	*/0.025	ns/0.522	*/0.014	ns/0.161	ns/0.521
	SB	*/0.039	ns/0.292	*/0.032	ns/0.284	ns/0.112	ns/0.084	ns/0.345	ns/0.408	ns/0.688	ns/0.839
	∑	*/< 0.001	*/0.001	*/< 0.001	ns/0.839	*/< 0.001	ns/0.223	ns/0.138	ns/0.619	ns/0.955	ns/0.624
Rosenberg		ns/0.99	ns/0.7689	ns/0.873	ns/0.974	ns/0.694	ns/0.639	*/0.014	ns/0.984	*/0.008	*/0.009
MSTS					ns/0.255				ns/0.828		

* Statistically significant

*ns* non significant

### Body image

Patients showed a reduced body image compared to control, with significantly lower scores in the overall score of the MBSRQ and in six different subscales (AE, FE, FO, HE, HO, SB) (Table 4).

### Self-esteem

Patients had a slightly lower RSE score compared to control, however, no statistically significant differences were observed (Table 4).

### Gender-specific QoL

Male patients scored significantly lower in subscales of the SF-36 assessing physical health compared to male control (Table 4). Female patients had lower scores of the SF-36 in additional physical health subscales than female controls (Table 4). No significant differences of QoL could be detected between male and female patients (Table 4).

### Gender-specific body image

In four different categories male patients had a lower overall body image than male controls (AE, FE, FO, HE) (Table 4). The body image of female patients was also inferior in the overall score as well as in five subscales (AE, FE, FO, GE and SB) compared to female control (Table 4).

Male patients had significantly lower scores of the MBSRQ in the AO category compared to female patients, whereas significantly lower scores for female patients were measured in two subscales (FE, FO) (Table 4).

### Gender-specific self-esteem

For both male and female patients no significant difference of self-esteem could be detected compared to same sex control (Table 4). Moreover, no significant difference between male and female patients was observed (Table 4).

### Tumor location-related QoL

A reduced QoL was detected for patients with tumors in the lower extremity compared to control with a significantly lower score of the SF-36 in the physical categories (Table 4).

Although patients with tumors in the upper extremity showed a reduced QoL in all categories of the SF-36 compared to control, it was only significant in one category (PF) (Table 4).

MH was significantly higher for patients with primary tumors located in the trunk, whereas PF was significantly lower in this group compared to control (Table 4).

QoL showed no significant differences between patients with tumors in the upper extremity and patients with tumors in the lower extremity (Table 4). Both groups had a significantly lower score in the MH subscale compared to patients with tumors located in the trunk (Table 4). Patients with tumors located in the trunk had a significantly higher QoL in two subscales (PF, RF) compared to patients with tumors located in the lower extremity (Table 4).

### Tumor location-related body image

Body image was reduced for patients with tumors in the lower extremity in all categories compared to control with significant differences in the overall score and five subscales (AE, FE, FO, GE and GO) (Table 4).

Patients with tumors in the upper extremity showed a reduced body image compared to control, although not significantly, except for one category (IO) where patients scored significantly higher (Table 4).

For the trunk group, a significantly reduced body image compared to control was detected in one subscale (AO) (Table 4). Similarly, this subscale was significantly reduced for the trunk group compared to the upper and lower extremity groups (Table 4).

A significant reduced body image could be seen in one subscale (IO) for patients with tumors located in the lower extremity compared to upper extremity (Table 4).

### Tumor location-related self-esteem

Patients with tumors in the trunk had a significantly higher self-esteem compared to control (Table 4). No significant difference of self-esteem was detected in the other tumor location groups compared to control. Moreover, compared to patients with tumors located in the trunk a significantly lower self-esteem was detected for the two other groups (Table 4).

### Treatment related QoL, self-esteem and body image

The use of radiotherapy for treatment did not influence QoL, body image and self-esteem of affected patients (Table 5).

**Table 5:** Treatment related QoL, body image and self-esteem

		Radiotherapy	No radiotherapy	Radiotherapy vs. no radiotherapy	Chemotherapy	No chemotherapy	Chemotherapy vs. no chemotherapy	Limb salvage	Amputation	Limb salvage vs. amputation
SF36	PF	78.57 (± 26.05)	63.41 (± 28.14)	ns/0.067	67.35 (± 27.45)	62.86 (± 34.98)	ns/0.754	66.48 (± 28.38)	40 (± 20.62)	*/0.041
	RF	67.86 (± 40.94)	59.15 (± 43.57)	ns/0.552	63.78 (± 42.71)	50 (± 43.3)	ns/0.454	60.8 (± 44.59)	40 (± 37.91)	ns/0.303
	BP	79 (± 27.71)	67.24 (± 28.08)	ns/0.171	70.84 (± 26.57)	63.43 (± 38.82)	ns/0.640	69.73 (± 29.91)	67 (± 14.73)	ns/0.741
	GH	71 (± 21.54)	67.15 (± 21.1)	ns/0.577	68.43 (± 20.07)	66.57 (± 27.84)	ns/0.869	67.86 (± 21.3)	63.6 (± 21.45)	ns/0.691
	V	66.79 (± 15.89)	56.71 (± 19.86)	ns/0.066	59.59 (± 17.04)	57.14 (± 32)	ns/0.849	58.07 (± 19.68)	55 (± 18.37)	ns/0.739
	SF	83.93 (± 29.18)	80.79 (± 26.38)	ns/0.764	83.42 (± 24.12)	71.43 (± 41.9)	ns/0.485	83.52 (± 24.67)	57.5 (± 42.94)	ns/0.482
	ER	80.95 (± 36.31)	78.86 (± 36.33)	ns/0.888	82.99 (± 33.42)	57.14 (± 46)	ns/0.195	81.06 (± 36.23)	73.33 (± 27.89)	ns/0.592
	MH	79.43 (± 11.73)	75.32 (± 18.72)	ns/0.376	76.9 (± 15.84)	74.29 (± 25.91)	ns/0.803	75.73 (± 16.87)	65.6 (± 20.9)	ns/0.348
MBSRQ	AE	3.45 (± 0.73)	3.61 (± 0.79)	ns/0.449	3.51 (± 0.76)	4.08 (± 0.75)	ns/0.097	3.52 (± 0.8)	3.71 (± 0.83)	ns/0.644
	AO	3.02 (± 0.71)	3.25 (± 0.64)	ns/0.296	3.17 (± 0.62)	3.36 (± 0.87)	ns/0.603	3.27 (± 0.62)	3.23 (± 0.71)	ns/0.906
	FE	3.83 (± 0.85)	3.56 (± 0.72)	ns/0.323	3.63 (± 0.77)	3.69 (± 0.71)	ns/0.297	3.55 (± 0.77)	3.88 (± 0.52)	ns/0.257
	FO	3.59 (± 0.65)	3.23 (± 0.94)	ns/0.134	3.36 (± 0.89)	3.16 (± 0.88)	ns/0.605	3.31 (± 0.91)	3.51 (± 0.68)	ns/0.579
	HE	3.8 (± 0.64)	3.56 (± 0.81)	ns/0.296	3.61 (± 0.74)	3.76 (± 0.99)	ns/0.704	3.55 (± 0.78)	3.63 (± 0.3)	ns/0.631
	HO	3.63 (± 0.67)	3.53 (± 0.58)	ns/0.648	3.54 (± 0.59)	3.68 (± 0.59)	ns/0.576	3.48 (± 0.6)	3.73 (± 0.37)	ns/0.231
	IO	3.37 ( ± 0.74)	3.09 (± 0.77)	ns/0.279	3.19 (± 0.76)	3.11 (± 0.89)	ns/0.840	3.19 (± 0.78)	2.96 (± 0.93)	ns/0.618
	SB	3.52 (± 0.56)	3.6 (± 0.81)	ns/0.675	3.49 (± 0.72)	4.22 (± 0.58)	*/0.019	3.55 (± 0.76)	3.91 (± 0.63)	ns/0.286
	∑	3.48 (± 0.4)	3.38 (± 0.45)	ns/0.473	3.4 (± 0.46)	3.5 (± 0.29)	ns/0.443	3.39 (± 0.46)	3.5 (± 0.25)	ns/0.408
RSE		24.71 (± 3.79)	23.63 (± 6.16)	ns/0.474	23.51 (± 5.73)	27.14 (± 3.39)	*/ 0.035	23.61 (± 5.62)	22 (± 7.11)	ns/0.646
MSTS		19 (± 7.96)	17.24 (± 7.4)	ns/0.537	17.59 (± 7.37)	17.71 (± 8.1)	ns/0.969	18.6 (± 6.95)	9 (± 5.57)	*/0.014

* Statistically significant

*ns* non significant

Regarding chemotherapy, a significantly reduced self-esteem for patients who were treated with chemotherapy was detected (Table 5).

A significantly decreased function was observed in amputated patients in the SF-36 and the MSTS compared to limb salvage procedure patients (Table 5).

## Discussion

The results of this study indicate that QoL of patients with primary malignant bone tumor is limited regarding physical functionality. An impaired body image of these patients, with a worse rating of appearance, function and health was also detected. However, patients’ self-esteem does not seem to be affected by primary bone sarcomas.

The aim of this study was to analyze whether or not and to which extent QoL, body image and self-esteem are affected in these patients. Therefore, affected patients were interviewed with four different self-administered questionnaires and compared to a healthy control cohort. As treatment and survival rates improve, QoL after treatment is gaining more and more importance. QoL is a complex construct, that can be affected by physical and psychological health as well as ones’ independence, social relationships and personal beliefs [18].

In this retrospective study physical health of patients with primary bone sarcoma had a negative effect on QoL, whereas psychological and social aspects of QoL were not influenced compared to healthy control. Similar findings were published in a study by Aksnes et al. in 2007, where the authors compared bone tumor patients with a healthy Norwegian population using the SF-36 [6]. In 57 bone sarcoma patients a significantly lower QoL in categories concerning physical health compared to normal population was found. Similar to the current study, categories depicting psychological health and social function were not influenced [6]. Other studies emphasized the trend that was found [8, 19]. Both studies investigated QoL in young patients with bone tumors using the SF-36 and observed that QoL is inferior regarding physical health compared to control. However, both could not find a psychological influence on QoL [8, 19]. However, both these studies included only young patients in their cohorts.

Another question which was addressed in the present study was how the two genders estimate their quality of life. Male patients showed higher scores in all categories of the SF-36 than female patients, albeit not significantly. Similar results were found in a study by Barrera et al. which revealed significantly higher QoL for male bone tumor patients in physical function, general health and social function compared to female patients [20]. These findings were also observed in other studies [21].

The expression “body-image” is the picture of your own body, which we form in our minds. It is influenced by ones’ cognitive perception, emotions and behavior [22]. The assessment of ones’ appearance and physical health can be affected due to cancer treatment [23]. Different tools including the MBSRQ can be used to assess the body image of patients [9, 24].

In this study, patients mentioned aesthetic and functional aspects as main concerns. Both male and female patients showed a significant lower body image in aesthetic and functional categories following sarcoma treatment compared to the controls. In the literature an increased, decreased or unaffected body image was observed in patients after cancer treatment [9, 10, 25]. However, these studies did not use the same tool compared to the current study.

The strength of the present study is that the body image is evaluated using a multidimensional score, which assesses appearance, fitness, health, illness evaluation and orientation together in primary bone sarcoma patients.

Comparing male and female patients, appearance orientation and health orientation were scored higher in female patients, although function evaluation and orientation were rated better in male patients. Some studies certify a better body image of male cancer patients [11, 26, 27], whereas other studies could not find a difference between the sexes [25, 28].

Patients were less satisfied with their appearance after they were treated with chemotherapy. This could be due to the side effects of chemotherapy. Münstedt et al. found a long-term descend of body image in female patients with leukemia after chemotherapy [29].

Self-esteem is a reflection of someone’s worth or value. In this study no significant difference in self-esteem of patients compared to control could be detected. However, RSE scores of patients were lower than healthy control. Consistent findings were published in a study by Langeveld et al. which evaluated self-esteem of 400 cancer survivors [11]. They found no significant difference between cancer patients and control, but detected lower self-esteem for female patients compared to male patients. This result could not be confirmed in this study.

Unlike other musculoskeletal diseases, such as rheumatoid arthritis and chronic back pain, bone sarcoma does not seem to have an influence on patient’s self-esteem [30, 31]. Patients with tumors located in the trunk had a higher self-esteem than patients with tumors located in the extremities, therefore, it can be stated that the location of the primary tumor seems to affect self-esteem. However, this could be due to the small sample size of patients with tumors in the trunk.

Maintaining the function of a limb is an important aspect which should be considered when deciding on a treatment.

Due to improving survival rates and the high proportion of young patients, surgical treatment has to preserve the function of the affected limb whenever possible. In this study, decreased functionality was detected for patients who received an amputation compared to patients who underwent limb-sparing procedures which was also found in other studies [32–35]. However, the function does not have to be impaired for amputated patients as shown by Nagarajan et al. published in 2004 illustrates [36]. It has to be pointed out that only five amputated patients were enrolled.

Different limiting factors have to be considered in this series. One limitation was the small amount of patients participating in this study. Furthermore it needs to be pointed out that the healthy controls were in average about ten years younger than the patients. This could pose a potential bias. But due to the rarity of primary malignant bone tumors and the fact that this study is a single-centered study, the enrolment of a higher number of patients was not possible. Another limiting factor is that not all patients where tested equally, as seven patients with no email address were questioned by telephone.

In summary, QoL and body image are impaired regarding functional and aesthetical aspects in patients with primary bone sarcoma. However, self-esteem does not seem to be affected by this condition or medical treatment. Patients can be encouraged about this at the time of diagnosis of a primary malignant bone tumor. Functional outcome after surgical treatment should be taken into account to achieve a good QoL for primary malignant bone tumor patients. This study highlights the importance of taking these factors into consideration when making clinical decision.
